# Supplementary material for: EnzML: multi-label prediction of enzyme classes using InterPro signatures
Source: BMC Bioinformatics. 2012 Apr 25;13:61. doi: 10.1186/1471-2105-13-61 (PMC3483700; doi:10.1186/1471-2105-13-61)
Supplement: Addtional file 5 — The Java code to format the data files, evaluate and predict. The file enzml_java_code.tar.gz contains the Java code used to format database data to ARFF and XML formats, to execute cross and train-test (jackknife) evaluations and to record evaluation results to database. More information is included in the readme.txt file and the Javadoc files. The code can be used with a MySQL database. To use a different database software, other JDBC drivers might be required. [file 1471-2105-13-61-S5.gz › java_code/enzml2011/doc/test/mulan/learn/traintest/TrainTestEvaluatorTest.html]

TrainTestEvaluatorTest


---


|  |  |  |
| --- | --- | --- |
| **Overview** \| **Package** \| **Class** \| **Use** \| **Tree** \| **Deprecated** \| **Index** \| **Help** | |  |
| **PREV CLASS**   **NEXT CLASS** | **FRAMES**    **NO FRAMES**     **All Classes** |  |
| SUMMARY: NESTED \| FIELD \| CONSTR \| METHOD | DETAIL: FIELD \| CONSTR \| METHOD |  |


---


## test.mulan.learn.traintest Class TrainTestEvaluatorTest

```
java.lang.Object
  junit.framework.Assert
      junit.framework.TestCase
          test.mulan.learn.traintest.TrainTestEvaluatorTest
```

**All Implemented Interfaces:**: junit.framework.Test

---

``` public class TrainTestEvaluatorTest extends junit.framework.TestCase ```

---

**Constructor Summary**

`TrainTestEvaluatorTest()`


| **Method Summary** | |
| --- | --- |
| `static java.util.Vector<uk.ac.ed.inf.utils.database.TableRow>` | `getEvaluationRows(TrainTestEvaluator evaluator)` |
| `static TrainTestEvaluator` | `getMulanTrainTestEvaluatorBRk1NN()` |
| `static uk.ac.ed.inf.utils.database.TableRow` | `testCheckTrainTestEvalRow(java.util.Vector<uk.ac.ed.inf.utils.database.TableRow> results)`             Executes generic tests (number of metrics etc.) on a table row containing evaluation results. |
| `void` | `testEvaluation1()` |
| `static void` | `testEvaluationResults1(uk.ac.ed.inf.utils.database.TableRow row)` |

**Methods inherited from class junit.framework.TestCase**

`countTestCases, getName, run, run, runBare, setName, toString`

**Methods inherited from class junit.framework.Assert**

`assertEquals, assertEquals, assertEquals, assertEquals, assertEquals, assertEquals, assertEquals, assertEquals, assertEquals, assertEquals, assertEquals, assertEquals, assertEquals, assertEquals, assertEquals, assertEquals, assertEquals, assertEquals, assertEquals, assertEquals, assertFalse, assertFalse, assertNotNull, assertNotNull, assertNotSame, assertNotSame, assertNull, assertNull, assertSame, assertSame, assertTrue, assertTrue, fail, fail, failNotEquals, failNotSame, failSame, format`

**Methods inherited from class java.lang.Object**

`equals, getClass, hashCode, notify, notifyAll, wait, wait, wait`

**Constructor Detail**

### TrainTestEvaluatorTest

```
public TrainTestEvaluatorTest()
```


**Method Detail**

### testEvaluation1

```
public void testEvaluation1()
```

---


### getEvaluationRows

```
public static java.util.Vector<uk.ac.ed.inf.utils.database.TableRow> getEvaluationRows(TrainTestEvaluator evaluator)
```

---


### getMulanTrainTestEvaluatorBRk1NN

```
public static TrainTestEvaluator getMulanTrainTestEvaluatorBRk1NN()
```

---


### testCheckTrainTestEvalRow

```
public static uk.ac.ed.inf.utils.database.TableRow testCheckTrainTestEvalRow(java.util.Vector<uk.ac.ed.inf.utils.database.TableRow> results)
```

:   Executes generic tests (number of metrics etc.) on a table row containing
    evaluation results.

    :   **Parameters:**: `results` - a vector of table rows **Returns:**: the first table row (for a train-test evaluation, only one row should be present)

---


### testEvaluationResults1

```
public static void testEvaluationResults1(uk.ac.ed.inf.utils.database.TableRow row)
```


---


|  |  |  |
| --- | --- | --- |
| **Overview** \| **Package** \| **Class** \| **Use** \| **Tree** \| **Deprecated** \| **Index** \| **Help** | |  |
| **PREV CLASS**   **NEXT CLASS** | **FRAMES**    **NO FRAMES**     **All Classes** |  |
| SUMMARY: NESTED \| FIELD \| CONSTR \| METHOD | DETAIL: FIELD \| CONSTR \| METHOD |  |


---
